# Postpartum Mental Health of Mothers in Fukushima: Insights From the Fukushima Health Management Survey’s 8-year Trends

**DOI:** 10.2188/jea.JE20210385

**Published:** 2022-12-05

**Authors:** Kayoko Ishii, Aya Goto, Hiromi Yoshida-Komiya, Tetsuya Ohira, Keiya Fujimori

**Affiliations:** 1 Department of Midwifery and Maternal Nursing, Fukushima Medical University School of Nursing, Fukushima, Japan; 2 Integrated Center for Science and Humanities, Fukushima Medical University, Fukushima, Japan; 3 Center for Gender-Specific Medicine, Fukushima Medical University, Fukushima, Japan; 4 Department of Epidemiology, Fukushima Medical University School of Medicine, Fukushima, Japan; 5 Department of Obstetrics and Gynecology, Fukushima Medical University School of Medicine, Fukushima, Japan; 6 Radiation Medical Science Center for the Fukushima Health Management Survey, Fukushima Medical University, Fukushima, Japan

**Keywords:** mother, depression, radiation, Great East Japan Earthquake, Fukushima nuclear accident

## Abstract

With the aim of monitoring the mental and physical health of mothers and children following the Fukushima nuclear accident and providing them with necessary care, we have been conducting an annual survey of expectant and nursing mothers since 2011. The Pregnancy and Birth Survey is a mail-in survey of about 15,000 individuals, with a response rate of approximately 50.0% each year. In addition, because respondents to a survey conducted in the immediate aftermath of the disaster showed a particularly high rate of depression, follow-up surveys have been conducted at 4 years after childbirth. Reviewing the results of surveys from FY 2011 through FY 2018, we found that the prevalence of depressive symptoms among mothers was highest in the survey after childbirth and decreased over time. Data of follow-up surveys showed that the prevalence of depression was lower than immediately after childbirth and then decreased over time. The proportion of mothers with radiation anxiety was higher among respondents in the FY 2011 follow-up than in the FY 2014 follow-up, indicating the prolonged impact of the nuclear accident, especially among those who gave birth immediately after the disaster. Characteristics of mothers who received telephone parenting counseling included first delivery, caesarean section, living in evacuation zones, not being able to receive medical examinations as scheduled, and having radiation anxiety. Continuous care should be provided to mothers who gave birth immediately after the nuclear accident, including routine perinatal care and parenting support, provision of information on radiation, and long-term monitoring of their wellbeing.

## INTRODUCTION

Pregnant women, as well as postpartum women and their children, are especially vulnerable to natural and technological disasters, particularly those who are highly exposed.^1^ In the aftermath of the Chernobyl nuclear accident, the impact on maternal mental health was reported to be one of the most serious public health consequences.^2^ After the 1979 Three Mile Island nuclear accident in the United States, pregnant women with a higher level of anxiety evaluated the health of their children as poorer.^3^ Likewise, pregnant women who have experienced natural disasters, such as severe hurricanes, are at a significantly higher risk of post-traumatic stress disorder and depression.^4^

The March 11, 2011 Great East Japan Earthquake was a complex disaster that consisted of an earthquake, a tsunami, and a nuclear accident, and immediately disrupted medical services and caused widespread and persistent radioactive contamination.^5^^,^^6^ Radiation anxiety was stronger within Fukushima Prefecture than outside, and mothers were more anxious than fathers in the prefecture.^7^ Fukushima Medical University launched the Fukushima Health Management Survey (FHMS) in fiscal year (FY) 2011, which was commissioned by the Fukushima Prefectural Government.^8^^,^^9^ The FHMS consists of the Basic Survey, which assesses the exposure doses of residents who were living in Fukushima Prefecture at the time of the disaster, and four detailed surveys: Thyroid Ultrasound Examinations for residents under 18 years old; a Comprehensive Health Checkup; a Mental Health and Lifestyle survey for residents in the evacuation zone; and the Pregnancy and Birth Survey for women who gave birth in Fukushima. The purpose of the Pregnancy and Birth Survey is to address anxieties associated with pregnancy, childbirth, and childrearing, as well as to provide counseling by midwives and public health nurses via telephone or email for respondents identified as in need of support.^10^

This paper offers a detailed account of depressive symptoms among mothers as well as the support provided to them, as shown in the results of the Pregnancy and Birth Survey. Our review aims to improve emergency preparedness targeting mothers with small children in future radiation-related disasters.

## METHODS

### Pregnancy and Birth Survey (survey after childbirth)

#### Survey design

##### Purpose

The purpose of the Pregnancy and Birth Survey is to address the anxiety that pregnant women and mothers in Fukushima Prefecture have and to provide necessary support by assessing their physical and mental health after the Great East Japan Earthquake and the accident at the Tokyo Electric Power Company’s Fukushima Daiichi Nuclear Power Station.

##### Target population

In the FY 2011–2018 surveys, 115,976 cases (FY 2011, 16,001; FY 2012, 14,516; FY 2013, 15,218; FY 2014, 15,125; FY 2015, 14,572; FY 2016, 14,154; FY 2017, 13,552; FY 2018, 12,838) satisfied one of the following conditions: received a Maternal and Child Health Handbook from one of the 59 municipal offices in Fukushima Prefecture between August 1 of the previous year and July 31 of the corresponding year, or received a Maternal and Child Health Handbook from an office outside Fukushima Prefecture, but received antenatal care and delivered their children in Fukushima Prefecture.

##### Procedure

The Pregnancy and Birth Survey of the FHMS has been conducted every year since the March 11, 2011 disaster, targeting women who have registered their pregnancies during a specified period each year. The pregnancy registration grants free access to antenatal care and well-child visits and is available to all pregnant women in Japan. Participants of the self-administered survey questionnaire were asked to respond by either mail or an online system that was available from FY 2016.^11^ The response period for the survey was January 2012 to March 2013 for FY 2011, December 2012 to November 2013 for FY 2012, December 2013 to December 2014 for FY 2013, November 2014 to December 2015 for FY 2014, November 2015 to December 2016 for FY 2015, November 2016 to December 2017 for FY 2016, November 2017 to December 2018 for FY 2017, and November 2018 to December 2019 for FY 2018.

##### Data items

The questionnaire items remained essentially the same in terms of content throughout the 8 years. The number of questions has changed only slightly. The survey items are maternal factors, including age, parity, family structure, depressive symptoms, and subjective health (FY 2012∼); disaster-related factors, including the region that issued the Maternal and Child Health Handbook (registered regions), evacuation status (FY 2012∼), and pregnancy intention (FY 2012∼); obstetric factors, including medical history, type of pregnancy, illness during pregnancy, delivery method, receipt of sufficient antenatal or delivery care; childrearing status, including infant feeding methods and the reasons for selecting these methods (FY 2011–2013) and maternal confidence (FY 2012∼); child-related factors, including the sex of the child, low birth weight, and congenital anomaly; and free comments.

The main indicator was maternal depressive symptoms as measured using a two-item screening tool for postpartum depression that was developed by Mishina et al.^12^ We asked, “During the past month, have you often felt down, depressed, or hopeless?” and “During the past month, have you often found little interest or pleasure in doing things?” We classified mothers who answered “Yes” to one or both of these questions as positive for depressive symptoms. It was reported that using the Edinburgh Postpartum Depression Scale (EPDS) as the standard, the sensitivity and specificity of the two-item screening tool was 88% and 76%, respectively.^12^ To perform a comparison with the national survey data in 2013 and 2017, we converted the two-item scores to EPDS scores using the positive and negative predictive value of the two-item measure reported by Mishina et al.^12^ It should be noted that an EPDS score of 9 or higher is classified as depression-positive in Japanese women (compared with the international baseline score of 13 or higher).

### Follow-up survey at 4 years after childbirth

#### Survey design

##### Purpose

Many of the respondents to the Pregnancy and Birth Survey at the time of the disaster tended to have depressive symptoms and wrote about serious issues in the free comment section of the survey form. Accordingly, a follow-up survey was implemented every year starting in FY 2015 targeting mothers 4 years after delivery, starting with those who responded to the questionnaire survey in FY 2011. We selected 4 years after delivery in order to provide a parenting support opportunity between the routinely conducted 36-month checkup and the pre-school checkup at about 5 years of age.

##### Target population

The target group consisted of 24,444 respondents (FY 2011, 7,252; FY 201, 5,602; FY 2013, 5,734; FY 2014, 5,856) of the FY 2011–2014 Pregnancy and Birth Surveys who were identified as alive along with their children through referral to municipalities (excluding those who miscarried, had their pregnancy terminated, or had a stillbirth).

##### Procedure

Self-administered survey questionnaires were mailed to eligible pregnant women at around 4 years after giving birth. An online system was launched in 2016. The response period for the survey was September 2015 to May 2016 for the FY 2011 follow-up, November 2016 to June 2017 for the FY 2012 follow-up, January 2018 to August 2018 for the FY 2013 follow-up, and January 2019 to August 2019 for the FY 2014 follow-up. In the follow-up survey, we provided the same necessary support after first assessing their physical and mental health, as in the first survey.

##### Data items

The survey items were simplified to six items: maternal confidence, two-item depression screening, subjective health, anxiety about radiation effects (eg, water, food, child’s outdoor play, child health, stigma, genetic influences), hospitalization of children, and mothers’ anxiety about their child (eg, physical and mental development, lifestyle, diseases). From the FY 2011 follow-up survey to the FY 2014 follow-up survey, no changes were made to any questionnaire items.

#### Support system for the Pregnancy and Birth Survey/Follow-up survey

##### Purpose

The aim of the support system was to alleviate the anxieties of Pregnancy and Birth Survey respondents who were judged to need consultation and support by having midwives and public health nurses provide consultation and support via telephone or email.

##### Target population deemed as requiring support

The target population consisted of respondents to the Pregnancy and Birth Survey or Follow-up survey who were judged to need consultation and support via telephone or email.

##### Criteria for support

Support was provided for respondents who satisfied at least one of the following criteria: 1) respondents who had two depressive symptoms; 2) respondents who, based on their free-written comments, appeared to be in a severely depressed mood, in need of support for child rearing, expressed concern about radiation exposure, complained of poor physical condition, or requested a direct substantial response or support. The same criteria were applied in follow-up surveys.

##### Support methods

After receiving the returned survey forms from the respondents, the responses are promptly checked and those who need support are identified. Midwives and public health nurses from the Radiation Medical Science Center for the Fukushima Health Management Survey provide counseling and support by phone sequentially. The support systems for the Pregnancy and Birth Survey and the Follow-up survey are similar. However, because of the difference in age of the children to be supported, support staff in the Pregnancy and Birth Survey received training on child growth and development, as well as care methods from birth to 1 year of age, before providing support. In the follow-up survey, the child was 4 years old, so the support staff received training on the developmental stages and challenges of 4-year-old children before providing support. In the primary survey, midwives provided the main support, while in the follow-up survey, the support provided by midwives was supplemented by clinical psychologists, who provided specialized support for developmental disorders, psychological problems, and other issues.

**Figure 1.**
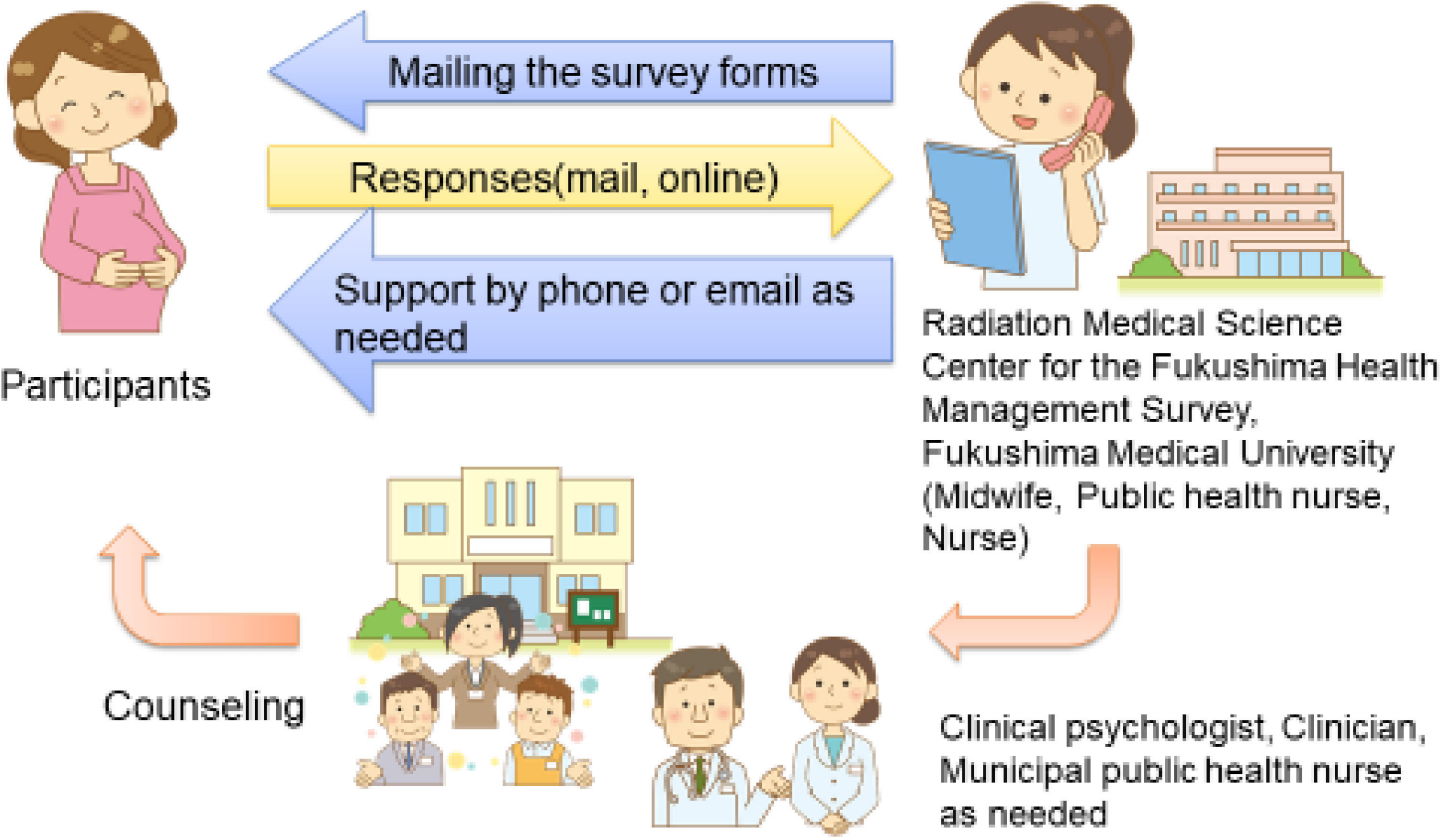
Flow of telephone consultation and support

In addition, a dedicated email address and a phone line for the survey are available for the respondents themselves to request a consultation. When a case requiring a more specialized response is identified through support by phone or email, the case is referred to specialized physicians or psychologists. For women for whom local support is found to be necessary, requests are made to the municipality where they reside to ask for further responses (Figure 1).

### Ethical considerations

The ethics committee of Fukushima Medical University approved this study (No. 1317, 2333). The aims of the survey were detailed in a cover letter that was attached to the questionnaires sent to all the participants. By responding to the survey, participants were considered to have consented to participation in the study.

## RESULTS

### Pregnancy and Birth Survey (survey after childbirth)

#### Survey results

##### Number of survey targets, number of responses, and response rate

**Figure 2.**
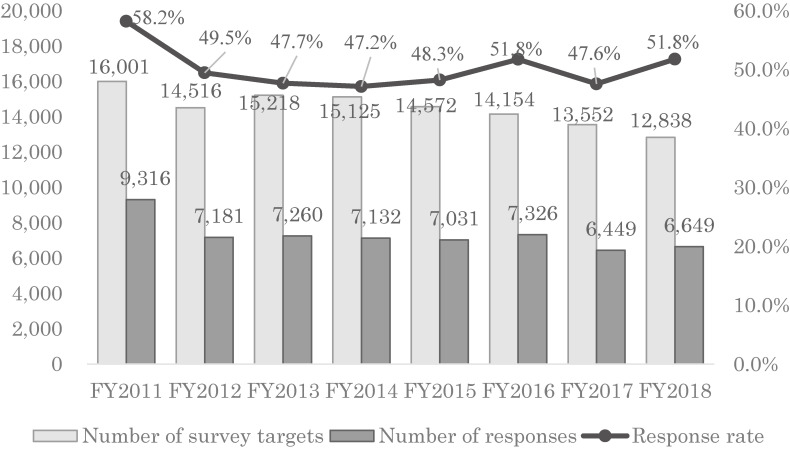
Number of survey targets, number of responses, and response rate (survey after childbirth)

In FY 2011, there were 16,001 survey targets, but in FY 2012, the number decreased substantially. In FY 2013, the target population recovered, but has been on a downward trend ever since. This trend is similar to that of the birth rate in the national survey. The response rate was highest in FY 2011 at 58.2% but has been consistent at around 50% ever since (Figure 2).^13^ The total number of responses from FY 2011–2018 was 58,344, and the response rate was 50.3%.

**Figure 3.**
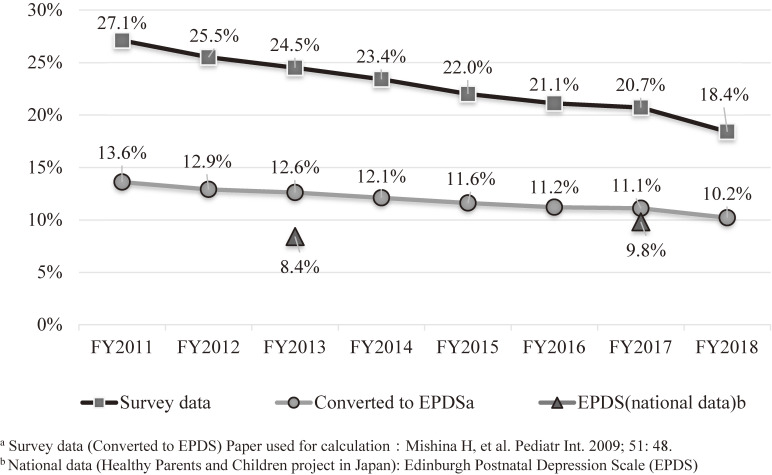
Proportion of maternal depressive symptoms (survey after childbirth)

Although the proportion of respondents reporting depressive symptoms was highest in FY 2011 at 27.1%, this had declined to 18.4% in FY 2018, showing a clear downward trend over the years (Figure 3).^13^ The overall depressive symptom rate for 8 years was 23.3%. To compare this with the national data, the estimated proportion of depression as measured by the EPDS in this survey in FY 2013 was 12.6%, which is higher than the 8.4% reported in the national survey data in 2013. However, in FY 2017, the estimated percentage of depression in this survey was 11.1%, which was similar to the 9.8% proportion from the national survey for the same year. The estimated percentage of cases of depression for the entire 8 years was 12.1%. In an analysis of the FY 2011 survey data (*n* = 8,196), the proportion of respondents with singleton live births who reported depressive symptoms was found to be 34.3% in the Sōsō coastal region near the nuclear power plant, which was higher than that in the coastal Iwaki region (24.2%), where radiation levels were relatively lower, or in the mountainous Aizu region (21.8%), far from the nuclear power plant. Moreover, it was found that, compared with mothers who did not switch facilities where they were scheduled to have antenatal checkups or deliver their babies, those who switched to other facilities were 1.3 times more likely to experience depression compared with those who did not switch.^14^ Furthermore, a higher proportion of depressive symptoms was observed among mothers who experienced miscarriage (41%, *P* < 0.01) or stillbirth (55%, *P* < 0.01) than among those who had a live birth (28%).^15^ Even using data from FY 2011–2018, the proportion of depressive symptoms in respondents who had a miscarriage (34%, *P* < 0.001) or stillbirth (50%, *P* < 0.001) was higher than in those who had a live birth (23%).

In the FY 2012 survey, the proportion of respondents indicating that they were still living as evacuees was 7.7%, but this decreased over the years to 1.7% by the time of the FY 2018 survey (FY 2012–2018, 4.3%). According to our analysis of the 2012 survey data, mothers who were issued a Maternal and Child Health Handbook in evacuation zones were 1.37 times more likely to experience depressive symptoms than those who lived outside evacuation zones (*P* = 0.001), and mothers still living as evacuees were 1.80 times more likely to be depressed than those who had not evacuated (*P* < 0.001). In nuclear families in particular, depressive symptoms were found in 55.1% of mothers still living as evacuees and not communicating often with their families, with whom they were not living together; this was higher than the 29.1% of mothers communicating often with their families with whom they were not living together (*P* = 0.002).^16^ In the analysis using data from FY 2012–2018, the percentage of mothers with depressive symptom among those who were still living as evacuees and often communicated with family members who lived elsewhere was 25.8%, compared with 51.7% for mothers who were still living as evacuees but were not communicating often with family members who lived elsewhere.

##### Maternal confidence

The percentage of mothers answering that they were not confident in child rearing ranged from 15.4% to 18.1% over the 7 years from FY 2012 to FY 2018, which did not indicate a significant change.^13^ The percentage of mothers who reported a lack of maternal confidence for the 7-year period FY 2012–2018 was 17.3%. In an analysis conducted using survey data from FY 2012/2013, it was found that, although depressive symptoms were associated with evacuation status and concerns about radiation, these were not associated with a lack of maternal confidence.^17^ In the data for the 7-year period FY 2012–2018, depressive symptoms were associated with evacuation status, but evacuation status was not associated with a lack of maternal confidence. There was a significant association between maternal confidence and depressive symptoms (Chi-square test, *P* < 0.001).

##### Feeding methods

In the questionnaire item concerning feeding methods before starting solids (FY 2011–2013), the proportion of mothers who answered breast milk only was 30.4% in 2011, 35.2% in 2012, and 36.6% in 2013, showing an increasing trend. In an analysis of survey data from FY 2011, the proportion of mothers who reported using baby formula (powdered milk) due to anxieties over radioactive contamination was 1.3 times higher (95% confidence interval [CI], 1.02–1.58) among mothers in evacuation zones compared with those outside evacuation zones, and was 1.3 times higher (95% CI, 1.09–1.57) among mothers who did not receive antenatal checkups as scheduled compared with those who did.^18^ In addition, to compare the coastal Sōsō region, which was strongly impacted by the nuclear accident, with the mountainous Aizu region, which was less affected, we divided the period from the occurrence of the disaster (March 11, 2011) to April 4, 2012 into four segments and found a tendency for the proportion of mothers answering baby formula only to increase over time (7.9%, 10.1%, 13.0%, 15.5%; *P* < 0.05). However, no differences were observed in terms of the growth of children at the time of the 1-month child health checkup.^19^

##### Pregnancy intention

The questionnaire item about intentions to have another baby were included in the survey starting in FY 2012. Although the percentage of mothers who answered that they were concerned about the effects of radiation as a reason for not wanting to have another pregnancy was 14.8% in 2012, this proportion trended downward over time, reaching 0.5% in FY 2018.^13^ For the 7-year period FY 2012–2018, this proportion was 4.1%. In an analysis using survey data from FY 2012–2014, it was found that although the proportion of multipara who wanted to have another baby had increased, the proportion of primipara who wished to do so was lower in 2013. This lack of intention was associated with using baby formula due to radiation concerns.^20^

##### Free comments

The highest proportion of free comments related to “concerns about the effects of radiation on the fetus or infant” was found in the FY 2011 survey (29.6%), but this had declined to 21.8% by the time of the FY 2018 survey. In contrast, a greater proportion of comments such as “request for improved parenting support services” and “consultation regarding child rearing” were observed starting in FY 2014 (see Table 1). In an analysis of survey data from FY 2011–2013, compared with respondents who did not write any free comments, the proportion of mothers who did were more likely to be at least 30 years old (61.5% wrote comments vs 54.4% who did not in FY 2011, 64.8% vs 57.4% in FY 2012, and 68.5% vs 60.8% in FY 2013; *P* < 0.001 for all items) and have depressive symptoms (34.8% wrote comments vs 22.1% who did not in FY 2011, 33.7% vs 23.0% in FY 2012, and 37.2% vs 22.7% in FY 2013; *P* < 0.001 for all items). Moreover, the main contents of the free comments shifted over time from radiation-related problems to complaints about their own poor physical and mental health.^21^

**Table 1.** Top three codes for mothers’ free comments

	Number 1	Number 2	Number 3
FY 2011	Radiation effect on fetus and infant	Information provision including survey results	Breast feeding and infant formula
FY 2012	Radiation effect on fetus and infant	Information provision including survey results	Complaints and suggestions about this survey
FY 2013	Complaints and suggestions about this survey	Radiation effect on fetus and infant	Mental and physical disorders
FY 2014	Improvement of childcare support	Child rearing counseling	Radiation effect on fetus and infant
FY 2015	Child rearing counseling	Improvement of childcare support	Improvement of medical services and physical care
FY 2016	Child rearing counseling	Improvement of childcare support	Mental and physical disorders
FY 2017	Child rearing counseling	Improvement of childcare support	Mental and physical disorders
FY 2018	Improvement of childcare support	Child rearing counseling	Mental and physical disorders

FY, fiscal year.

#### Support results

##### Number of respondents requiring support

**Figure 4.**
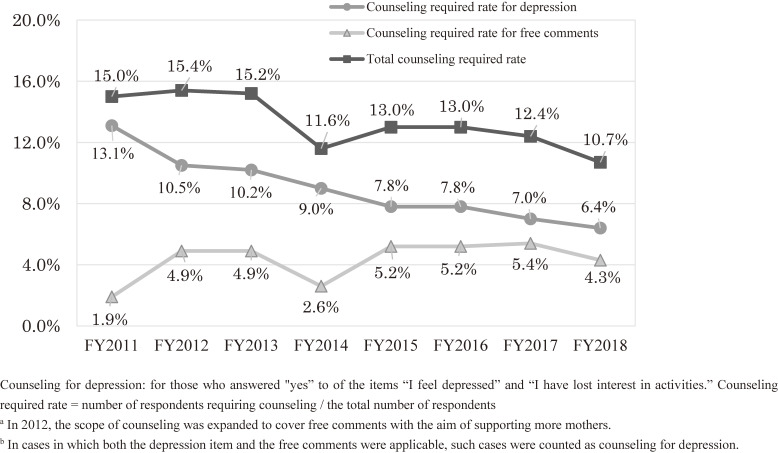
Change in the rate of respondents requiring counseling (survey after childbirth)

Every year, telephone support was provided to nearly 1,000 respondents. The proportion of those receiving telephone support was 15.0% in FY 2011, but this had decreased to 10.7% by FY 2018. For the 8-year period FY 2011–2018, this proportion was 13.4%. Although the proportion of support based on the content of the free comments has been expanding, the overall proportion of support requirements is trending downward (Figure 4).

##### Characteristics of mothers who required telephone counseling

The proportion of support-requiring mothers who had depressive symptoms was 9.2% for the 8-year period FY 2011–2018, but this decreased by almost half, from 13.1% in FY 2011 to 6.4% in FY 2018 (Figure 4). In an analysis of survey data from FY 2011, depressive symptoms accounted for 87.3% of the reasons for requiring telephone support. It was found that mothers who needed telephone support were significantly more likely than those who did not to be in evacuation zones (16.2% vs 11.8%; *P* < 0.001), to be unable to continue antenatal care (24.0% vs 18.1%; *P* < 0.001), to have bottle-fed because of radiation concerns (21.8% vs 12.4%; *P* < 0.001), to be primiparous (32.9% vs 26.5%; *P* < 0.001), to have had a cesarean section (23.2% vs 19.4%; *P* < 0.01), or to have given birth to a child with congenital anomalies (4.6% vs 2.3%; *P* < 0.001).^22^

##### Consultation contents

**Figure 5.**
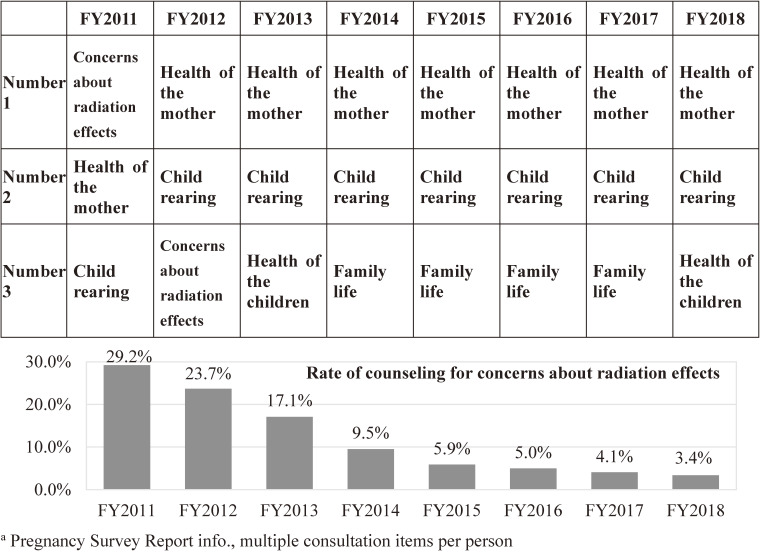
Top three codes for contents of telephone counseling (survey after childbirth)

The primary contents of telephone consultations were “physical and mental health of mothers,” “child-rearing (life with children),” and “physical and mental health of children,” which accounted for 43.6%, 34.7%, and 15.1%, respectively, of the total for FY 2011–2018. The proportion of the primary contents of telephone consultations shifted over the years. In FY 2011, in the immediate aftermath of the disaster, the most frequent consultation was related to “concerns about radiation effects” (29.2%), but this topic declined over the years to just 3.4% in FY 2018. Since FY 2012, a large proportion of consultations have focused on the “physical and mental health of mothers” and “child-rearing (life with children)” (Figure 5).

### Follow-up survey at 4 years after childbirth

#### Survey results

##### Number of survey targets, number of responses, and response rate

**Figure 6.**
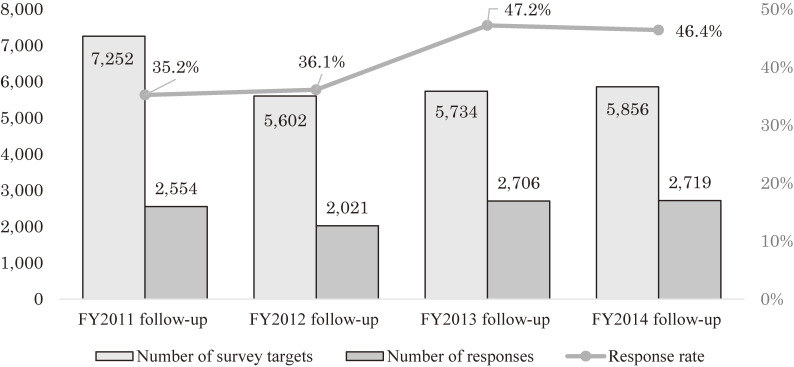
Number of survey targets, number of responses, and response rate (4th year follow-up survey)

Although the response rate to the follow-up survey was 35.2% for the FY 2011 follow-up, which was lower than that of the primary survey, this increased to 46.2% for the FY 2014 follow-up, which was higher than that in the primary year (Figure 6). The total response rate for the FY 2011–2014 follow-up surveys was 40.9%.

##### Maternal depressive symptoms

**Figure 7.**
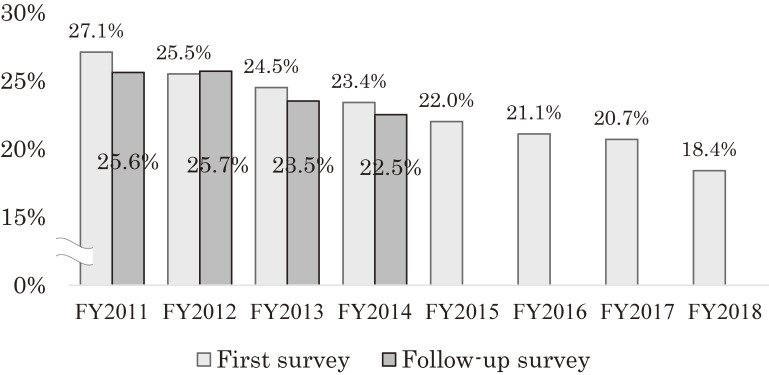
Proportion of maternal depressive symptoms (survey after childbirth vs 4th year follow-up survey)

The proportion of mothers who were found to have depressive symptoms in the 4-year follow-up survey was generally lower than in the primary survey (FY 2011, primary 27.1%, follow-up 25.6%; FY 2012, primary 25.5%, follow-up 25.7%; FY 2013, primary 24.5%, follow-up 23.5%; FY 2014, primary 23.4%, follow-up 22.5%; FY 2011–2014 primary 25.3%, follow-up 24.2%). This proportion has shown a downward trend over the years. However, it was found that depressive symptoms were higher among respondents to the follow-up survey than among respondents to the primary survey in the same year (FY 2011 follow-up, 25.6% vs FY 2015 primary, 22.0%; FY 2012 follow-up, 25.7% vs FY 2016 primary, 21.1%; FY 2013 follow-up, 24.5% vs FY 2017 primary, 20.7%; FY 2014 follow-up, 22.5% vs FY 2018 primary, 18.4%). Mothers who gave birth immediately after the disaster showed a tendency to still be depressed even after 4 years (Figure 7).

##### Maternal confidence

Since 2012, we have asked questions about maternal confidence in child rearing, as in the primary survey. The proportion of mothers who reported lacking confidence was found to be almost the same in the follow-up survey as in the primary survey 4 years earlier (FY 2012 follow-up, 18.2% vs primary, 15.4%; FY 2013 follow-up, 16.7% vs primary, 17.5%; FY 2014 follow-up, 17.7% vs primary, 16.6%; FY 2012–2014 follow-up, 18.8% vs primary, 18.3%).^23^

##### Anxiety about radiation effects

**Figure 8.**
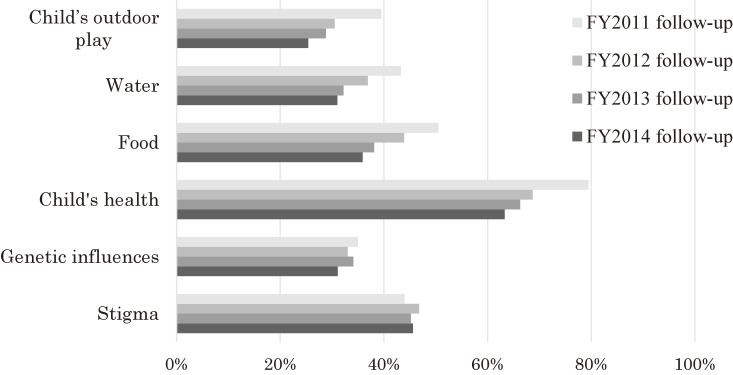
Concerns about the effects of radiation (4th year follow-up survey)

Although the proportion of respondents who checked one or more of the six items concerning anxiety about the effects of radiation was 94% in the follow-up to the FY 2011 survey, this gradually decreased to 85% in the follow-up to the FY 2014 survey (FY 2011–2014 follow-up, 89%). The most commonly checked item was “child’s health.” While the proportions of concerns about the child’s outdoor play, water, food, and the child’s health have been decreasing sharply, concerns about genetic influences have been decreasing slowly. Of note, concerns about the stigma of radiation exposure have not changed much (Figure 8). In an analysis of data from the follow-up to the FY 2011 survey, 41.2% of mothers felt anxiety due to prejudice and discrimination associated with exposure to radiation. Such concerns were found to be significantly associated with maternal age at the time of pregnancy (adjusted odds ratio [aOR] 1.03, *P* = 0.001), depressive symptoms (aOR 1.34, *P* = 0.024), not attending antenatal care as scheduled (aOR 1.40, *P* = 0.003), and post-quake illness (aOR 1.36, *P* = 0.006).^24^

##### Mothers’ anxiety about their children

The proportion of respondents who checked at least one item related to concerns about their children were 70.8% in the follow-up to the FY 2011 survey, 66.9% in the FY 2012 follow-up, 61.2% in the FY 2013 follow-up, and 63.4% in the FY 2014 follow-up (FY 2011–2014 follow-up, 65.4%). “Physical and mental development” and “diseases” ranked highly as specific concerns. Although the proportion of respondents concerned about “physical and mental development” did not change significantly (56.1% in the follow-up to the FY 2011 survey, 56.9% in the FY 2012 follow-up, 57.4% in the FY 2013 follow-up, and 56.9% in the FY 2014 follow-up), those concerned about “diseases” were found to decline over time (57.6% in the follow-up to the FY 2011 survey, 45.5% in the FY 2012 follow-up, 40.4% in the FY 2013 follow-up, and 38.7% in the FY 2014 follow-up).^23^

##### Free comments

The proportion of respondents who wrote comments in the free comment section was highest in the follow-up to the FY 2011 survey (15.0%). Thereafter, a declining trend was observed over time, to 9.2% in the FY 2012 follow-up, 7.7% in the FY 2013 follow-up, and 7.3% in the FY 2014 follow-up. When the contents of the comments were categorized, it was found that although “concerns about the effects of radiation on the fetus or infant” were most common in the follow-up to the FY 2011 survey (13.8%), a declining trend was observed over time, to 12.4% in the FY 2012 follow-up, 11.5% in the FY 2013 follow-up, and 7.1% in the FY 2014 follow-up. The most common and second-most common contents in the free comment section from the FY 2012 follow-up to the FY 2014 follow-up were “positive comments about this survey” and “opinions or complaints about this survey.”

#### Support results

##### Number of mothers requiring support

**Figure 9.**
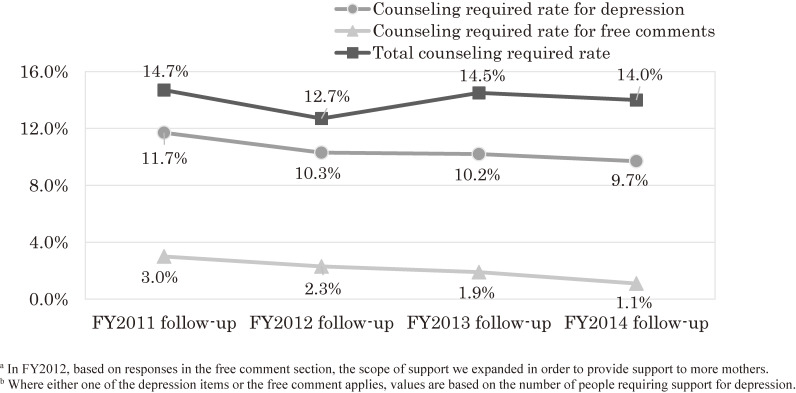
Change in the rate of respondents requiring counseling (4th year follow-up survey)

In the follow-up surveys, the proportion of support-requiring mothers according to depressive symptoms showed a declining trend, from 11.7% in the FY 2011 follow-up to 10.3% in the FY 2012 follow-up, 10.2% in the FY 2013 follow-up, and 9.7% in the FY 2014 follow-up (FY 2011–2014 follow-up, 10.5%). After the follow-up to the FY 2013 survey, support began to be provided to mothers who wrote specific concerns in spaces other than the free comment section (Figure 9).

##### Consultation contents

**Figure 10.**
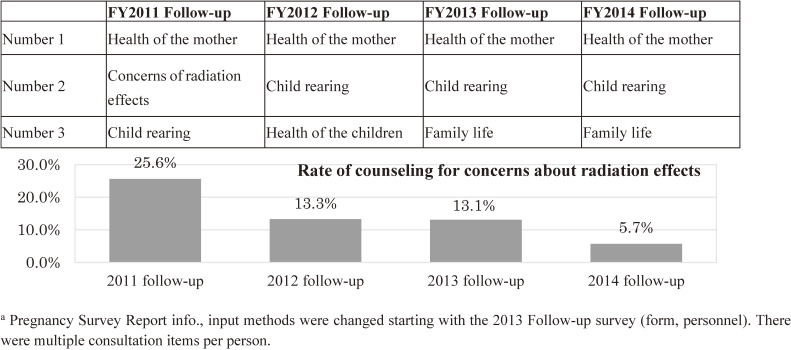
Top three codes for contents of telephone counseling (4th year follow-up survey)

In the follow-up surveys, “complaints about their own poor physical and mental health” consistently ranked highest from the FY 2011 follow-up to the FY 2014 follow-up, whereas the proportion of consultations for “concerns about the effects of radiation” has declined over time (Figure 10) (FY 2011–2014 follow-up, 15.1%).

## DISCUSSION

### Changes over time in maternal depressive symptoms (survey after childbirth)

In the primary survey for FY 2011, the proportion of respondents reporting depressive symptoms (a total score of 9 or greater when converted to the EPDS) was 13.6%. One of studies conducted by the Ministry of the Environment as part of the Japan Environment and Children’s Study compared mental health among mothers in Fukushima Prefecture with those in 13 other prefectures across Japan that were not affected by the 2011 disaster. Based on this, it was found that 16.1% of mothers had depressive symptoms (a total score of 9 or more when on the EPDS) in FY 2011 in Fukushima Prefecture compared with 11.5% of mothers in other prefectures.^25^ Because there have been no studies comparing Miyagi, Iwate, and Fukushima—three prefectures with different degrees of damages caused by tsunami, earthquake, and the nuclear accident—we cannot estimate differences in impacts on maternal mental health according to the type of disaster. Nevertheless, according to a FY 2011 survey conducted in Miyagi and Iwate Prefectures, which were heavily affected by the earthquake and tsunami and somewhat affected by the nuclear accident, the proportion of mothers with an EPDS score of 9 or more was between 21.3% and 21.5%.^26^^–^^28^ In Iwate Prefecture, it was reported that although the proportion of mothers with EPDS scores of 9 or more prior to the disaster had been 9.9%, this value was 31.4% among those who gave birth within 3 months of the disaster.^29^ Evidence from the 2011 disaster indicates that the proportion of depressive symptoms among mothers rose sharply in affected areas, especially in its immediate aftermath.

In the wake of Hurricane Katrina in the United States in 2005 as well as the 1988 earthquake in Armenia, the proportion of mothers who screened positive for postpartum depression on the EPDS ranged from 13% to 28%.^30^^–^^34^ These reports showed that mothers affected by disaster often experienced postpartum depression, regardless of the type of disaster. Harville et al^1^ reported in 2010 that they had searched publications published in the previous 10 years that more broadly covered the effects of disaster on perinatal health. Their work included terrorist attacks, environmental and chemical disasters, and natural disasters, and found that severity of exposure was a major predictor of mental health issues among postpartum women.

Although mothers with depressive symptoms accounted for a higher proportion of those with anxiety about the effects of radiation on their children, it is encouraging that the proportion of free comments regarding the effects of radiation on their child decreased significantly over time along with a reduction of depressive symptoms. Similar to these trends, previous studies on the counseling records of mothers with young children in the relatively radioactive contaminated area of Fukushima City revealed that a lack of maternal confidence, which was associated with risk perception toward radiation exposure, increased in the disaster year but decreased the year after.^35^ In the Mental Health and Lifestyle Survey, which is section of the FHMS targeting residents living in evacuation zones, psychological distress was measured longitudinally using the Kessler Screening Scale for Psychological Distress. In this 7-year survey, a gradual improvement was observed,^36^ which is consistent with the results of our own survey. However, even 7 years after the disaster, the proportion of individuals exhibiting depressive symptoms in evacuation zones remained higher than in other areas, indicating the possibility that anxieties about radiation may persist for a long time. Accordingly, we believe that mental health monitoring and support were still needed by mothers living in evacuation zones.

### Associated factors of maternal depressive symptoms

Existing data from the FHMS showed that factors related to depressive symptoms included living in evacuation zones near the nuclear power plant, remaining in a state of evacuation, and unplanned switching of obstetrical facilities. Risk factors for poor mental health reported in other studies included destruction of homes, exposure to the tsunami, loss of employment, and dissatisfaction with one’s living conditions.^26^^–^^29^ Severity of disaster exposure is another factor associated with depressive symptoms, as reported in overseas studies. According to a review by Harville et al,^1^ severity of exposure to a disaster is a major predictor of mental health problems in postpartum women. For example, the proportion of mothers with depressive symptoms, as defined by the EPDS, was 28.7% for tsunami survivors, 23.9% for evacuees, and 25.6% for those without a job.^28^^,^^37^ In addition, data from both FY 2012 and the 7-year period FY 2012–2018 showed that mothers who communicated with their families during the evacuation had lower depressive symptoms than those who did not. Therefore, cooperation among communities is needed so that mothers can communicate with their families even during an evacuation.

Well-known factors frequently associated with postpartum depression in a normal setting, both in Japan and overseas, include obstetrical history^22^^,^^38^ (eg, maternal age,^28^^,^^31^ first delivery, miscarriage and stillbirth,^15^ cesarean section, and presence of congenital anomalies in the child), as well as economic status,^27^^,^^32^^,^^34^^,^^38^^,^^39^ partner support,^30^^,^^33^^,^^40^ family relationships,^38^ lack of childcare support,^27^ negative feelings toward pregnancy,^25^ and a lack of maternal affection toward their child.^25^^,^^38^ Miscarriage and stillbirth were shown to be associated with depressive symptoms both in the primary survey for FY 2011,^15^ and in the 8-year period FY 2011–2018. This suggests that the results of this previous study were applicable to the whole study period. In the survey data from FY 2012/2013, disaster-related factors (evacuation, radiation anxiety) were associated with depressive symptoms but not with maternal confidence,^17^ and the same results were obtained for the 7-year period FY 2012–2018. Maternal confidence and depression tended to be associated with each other, but each had different associations with disaster impacts, as seen in the present study. More precisely, maternal confidence was not affected by disaster-related stressors, indicating resilience in parenting. On the other hand, we confirmed that mothers’ experiences and concerns in the aftermath of nuclear disaster were associated with depressive symptoms, highlighting the importance of recognizing mothers as strong actors during recovery from a disaster and, at the same time, recognizing their need for continuous mental support.

In the FHMS, depressive symptoms were further associated with increased use of baby formula and decreased desire to have another baby. A prospective cohort study conducted after Hurricane Katrina reported that maternal mental health influenced the child’s temperament.^31^ In the aftermath of environmental disasters worldwide, there has been concern regarding maternal mental health and the negative impacts on child development.^1^ After the 1986 Chernobyl disaster in Ukraine, the pregnancy period was shortened in women with mental and physical anxiety.^41^ There was also an increase in speech and emotional disorders in children due to adjustment disorders associated with parental anxiety.^42^ Women, especially those caring for young children, are one of the groups at the highest risk of mental and physical health problems.^1^ Mothers affected by a disaster need appropriate support because of the expected long-term effects on the child as well as themselves.

### Changes over time in maternal depressive symptoms (follow-up survey at 4 years after childbirth)

In the follow-up surveys, it was found that the proportion of mothers with depressive symptoms in the fourth year after delivery had decreased. However, given that the proportion of mothers harboring anxiety about radiation was higher in the FY 2011 follow-up than in the FY 2014 follow-up, it seemed that post-disaster psychological impacts persisted, especially among mothers who delivered immediately after disaster. The 20th anniversary report of the Chernobyl Forum indicated that many people who were exposed to radiation at the time of the accident continued to have a high proportion of depression and post-traumatic stress disorder 20 years later.^2^

Mothers are most concerned about their children’s health. However, with the passage of time, anxiety about radiation is gradually decreasing as mothers observe that their children’s health status has not been altered because of the nuclear accident. In contrast, anxieties about prejudicial attitudes have not abated. Immigrants who left Russia due to the effects of Chernobyl nuclear accident were more likely to experience a sense of prejudice compared with those who left for other reasons.^43^ In addition, in the Fukushima Mental Health and Lifestyle Survey, which targeted residents in evacuation zones, the proportion of respondents who felt that “the possibility is very high” with respect to the late health effects or genetic effects of radiation decreased, but the proportion of those who felt that “the possibility is high” did not.^36^ Difficulty remains for alleviating people’s concerns toward an uncertain risk factor and stigma.

### Support system

Mothers receiving telephone counseling in our survey were more likely to be first-time mothers, use baby formula due to anxieties over radiation, live in evacuation zones, and experience unforeseen circumstances, such as not being able to attend antenatal checkups as scheduled in the aftermath of the disaster. During pregnancy, women experience physical and psychological changes, making them more vulnerable to disasters. According to intervention research conducted overseas, a study on Hurricane Isaac implemented a needs assessment toolkit (Reproductive Health Assessment After Disasters Toolkit) and reported the importance of providing comprehensive disaster management in order to reduce fragmentation in health care systems.^44^ Another study after Hurricane Katrina described the common practice of complementary alternative therapy.^45^ Even at the time of the crisis, support is required for well-known high-risk factors, such as first-time pregnancies, cesarean sections, and congenital anomalies in the child. An Australian study conducted after a flood disaster reported the potential positive impacts of continuous care by an assigned midwife.^46^ After Hurricane Katrina, New Orleans Healthy Start, a safety-net community program for mothers socially at risk, provided social services, education, and referrals to families in need.^47^ Therefore, we recommend implementation of comprehensive needs assessment (eg, Reproductive Health Assessment After Disaster Toolkit), and correspondingly, provision of comprehensive support (eg, Healthy Start) and continuous professional care (eg, midwifery group practice care). Forms of support that are required include inter-regional collaboration, assessment of the needs of individuals by medical professionals, provision of accurate information to reduce radiation anxiety, routine care for expectant and nursing mothers, and provision of childcare consultations to restore a sense of normalcy in extraordinary situations. The contents of telephone consultations over the 8-year period FY 2011–2018 included consultations on not only “concerns about radiation effects” but also “physical and mental health of mothers,” “child-rearing (life with children),” and “physical and mental health of children”. Ordinary care was also considered to be important following a disaster. Unfortunately, in this study, we did not evaluate whether the mental health situation of the mothers who received the support improved or not. However, this support system enabled us to provide support to a wide range of mothers who may not have been able to seek support on their own, because the support staff telephoned those who were prone to depression. In addition, with the consent of the subject, their information was provided to the local government official where the subject lived, which enabled us to refer them with more careful support.

### Limitations and future tasks of the Pregnancy and Birth Survey

There were two major limitations of the present study. First, because the response rate in this survey was only about half, its findings cannot be said to reflect the circumstances of mothers with small children in Fukushima Prefecture as a whole. Second, some well-known factors of postpartum depression, including socioeconomic and support network factors, were not included in the survey questionnaires of the present study. The implications for future crisis management include the need for more detailed consideration of disaster exposure and household conditions when planning post-disaster epidemiological studies. The strengths of this study lie in the fact that this research offers a rare example of a survey that has investigated the impact of radiation exposure of an extended time scale of 10 years, as well as its use of a follow-up survey 4 years later.

### Conclusion

The findings of our prefecture-wide study have shown that mothers in Fukushima were likely to experience depression in the immediate aftermath of the Great East Japan Earthquake, and the prevalence was higher than in the national data or in unaffected areas. On a positive note, this tendency has decreased over time. Mothers who gave birth soon after the disaster were more likely to continue to have depression 4 years later compared with women who gave birth that year. This finding suggests the possibility of a lasting impact of the disaster on the mental health of women who were expectant or nursing mothers at the time. Accordingly, continuous support is necessary for mothers who gave birth in the immediate aftermath of the disaster.

## References

[1] Harville E, Xiong X, Buekens P. Disasters and perinatal health: a systematic review. Obstet Gynecol Surv. 2010;65:713–728. 10.1097/OGX.0b013e31820eddbe 21375788 PMC3472448

[2] Bromet EJ, Havenaar JM, Guey LT. A 25 year retrospective review of the psychological consequences of the Chernobyl accident. Clin Oncol. 2011;23:297–305. 10.1016/j.clon.2011.01.501 21330117

[3] Houts PS, Tokuhata GK, Bratz J, Bartholomew MJ, Sheffer KW. Effect of pregnancy during TMI crisis on mothers’ mental health and their child’s development. Am J Public Health. 1991;81:384–386. 10.2105/AJPH.81.3.384 1994750 PMC1405026

[4] Xiong X, Harville EW, Mattison DR, . Hurricane Katrina experience and the risk of post-traumatic stress disorder and depression among pregnant women. Am J Disaster Med. 2010;5(3):181–187. 10.5055/ajdm.2010.0022 20701175 PMC3501144

[5] Hachiya M, Tominaga T, Tatsuzaki H, Akashi M. Medical management of the consequences of the Fukushima nuclear power plant incident. Drug Dev Res. 2014;75:3–9. 10.1002/ddr.21161 24648044

[6] Fujimori K, Nomura Y, Yasuda S, . Obstetrical care in Fukushima Prefecture: obstetrical care and pregnancy trends immediately after the disaster. Shusanki Igaku. 2012;42:303–306 [in Japanese].

[7] Tateno S, Yokoyama HM. Public anxiety, trust, and the role of mediators in communicating risk of exposure to low dose radiation after the Fukushima Daiichi Nuclear Plant explosion. JCOM. 2013;12:A03. 10.22323/2.12020203

[8] Yasumura S, Hosoya M, Yamashita S, ; Fukushima Health Management Survey Group. Study protocol for the Fukushima Health Management Survey. J Epidemiol. 2012;22:375–383. 10.2188/jea.JE20120105 22955043 PMC3798631

[9] Yasumura S, Ohira T, Ishikawa T, . Achievements and current status of the Fukushima Health Management Survey. J Epidemiol. 2022;32(Suppl 12):S3–S10. 10.2188/jea.JE20210390 PMC9703928 36464298

[10] Fujimori K, Kyozuka H, Yasuda S, ; Pregnancy and Birth Survey Group of the Fukushima Health Management Survey. Pregnancy and birth survey after the Great East Japan Earthquake and Fukushima Daiichi Nuclear Power Plant accident in Fukushima Prefecture. Fukushima J Med Sci. 2014;60:75–81. 10.5387/fms.2014-9 25030719

[11] Nakano H, Ishii K, Goto A, . Development and implementation of an Internet survey to assess community health in the face of a health crisis: data from the Pregnancy and Birth Survey of the Fukushima Health Management Survey, 2016. Int J Environ Res Public Health. 2019;16:1946. 10.3390/ijerph16111946 31159365 PMC6603910

[12] Mishina H, Hayashino Y, Fukuhara S. Test performance of two-question screening for postpartum depressive symptoms. Pediatr Int. 2009;51:48–53. 10.1111/j.1442-200X.2008.02659.x 19371277

[13] Radiation Medical Science Center for the Fukushima Health Management Survey. Materials and minutes of prefectural oversight committee meetings. 37th report (Feb 13, 2020). http://kenko-kanri.jp/en/health-survey/document/pdf/37_13Feb2020.pdf; 2020 Accessed 7.17.2021.

[14] Goto A, Bromet EJ, Fujimori K; Pregnancy and Birth Survey Group of Fukushima Health Management Survey. Immediate effects of the Fukushima nuclear power plant disaster on depressive symptoms among mothers with infants: a prefectural-wide cross-sectional study from the Fukushima Health Management Survey. BMC Psychiatry. 2015;15:59. 10.1186/s12888-015-0443-8 25885267 PMC4393633

[15] Yoshida-Komiya H, Goto A, Yasumura S, Fujimori K, Abe M; Pregnancy and Birth Survey Group of Fukushima Health Management Survey. Immediate mental consequences of the Great East Japan Earthquake and Fukushima Nuclear Power Plant accident on mothers experiencing miscarriage, abortion, and stillbirth: the Fukushima Health Management Survey. Fukushima J Med Sci. 2015;61(1):66–71. 10.5387/fms.2014-33 26063510 PMC5131602

[16] Ohta M, Hattori S, Arai M, . Lives and depressive tendencies of pregnant women and nursing mothers evacuated due to the 2011 Great East Japan Earthquake: an investigation based on the Pregnancy and Birth Survey of the FY 2012 Fukushima Health Management Survey. J Jpn Matern Infant Care Assoc. 2019;12:21–31 [in Japanese].

[17] Goto A, Bromet EJ, Ota M, Ohtsuru A, Yasumura S, Fujimori K; Pregnancy and Birth Survey Group of the Fukushima Health Management Survey. The Fukushima Nuclear Accident Affected Mothers’ Depression but Not Maternal Confidence. Asia Pac J Public Health. 2017 Mar;29(2_suppl):139S–150S. 10.1177/1010539516684945 28330405

[18] Ishii K, Goto A, Ota M, Yasumura S, Abe M, Fujimori K; Pregnancy and Birth Survey Group of the Fukushima Health Management Survey. Factors associated with infant feeding methods after the nuclear power plant accident in Fukushima: data from the Pregnancy and Birth Survey for the Fiscal Year 2011 Fukushima Health Management Survey. Matern Child Health J. 2016 Aug;20(8):1704–1712. 10.1007/s10995-016-1973-5 27028325 PMC4935738

[19] Kyozuka H, Yasuda S, Kawamura M, . Impact of the Great East Japan Earthquake on feeding methods and newborn growth at 1 month postpartum: results from the Fukushima Health Management Survey. Radiat Environ Biophys. 2016;55:139–146. 10.1007/s00411-016-0636-7 26875100 PMC4840221

[20] Goto A, Tsugawa Y, Fujimori K. Factors associated with intention of future pregnancy among women affected by the Fukushima Nuclear Accident: Analysis of Fukushima Health Management Survey Data from 2012 to 2014. J Epidemiol. 2019 Aug 5;29(8):308–314. 10.2188/jea.JE20180015 30555116 PMC6614079

[21] Ito S, Goto A, Ishii K, Ota M, Yasumura S, Fujimori K; Pregnancy and Birth Survey Group of the Fukushima Health Management Survey. Fukushima mothers’ concerns and associated factors after the Fukushima nuclear power plant disaster: analysis of qualitative data from the Fukushima Health Management Survey 2011–2013. Asia Pac J Public Health. 2017 Mar;29(2_suppl):151S–160S. 10.1177/1010539516684533 28330402

[22] Ishii K, Goto A, Ohta M, Yasumura S, Fujimori K. Content and characteristics of mothers receiving telephone counseling following the Fukushima Nuclear Power Plant accident - data from the pregnancy and birth survey of the Fukushima health management survey, 2011-. Jpn Soc Matern Health. 2017;57:652–659 [in Japanese].

[23] Radiation Medical Science Center for the Fukushima Health Management Survey. Materials and minutes of prefectural oversight committee meetings. 37th report (Feb 13, 2020). https://www.pref.fukushima.lg.jp/uploaded/attachment/369429.pdf (In Japanese); 2020 Accessed 7.17.2021.

[24] Ito S, Goto A, Ishii K, . Overview of the Pregnancy and Birth Survey section of the Fukushima Health Management Survey: focusing on mother’s anxieties toward radioactive exposure. J Natl Inst Public Health. 2018;67(1):59–70. 10.20683/jniph.67.1_59

[25] Kuroda Y, Goto A, Koyama Y, ; Japan Environment and Children’s Study (JECS). Antenatal and postnatal association of maternal bonding and mental health in Fukushima after the Great East Japan Earthquake of 2011: The Japan Environment and Children’s Study (JECS). J Affect Disord. 2021;278:244–251. 10.1016/j.jad.2020.09.021 32971317

[26] Nishigori H, Sugawara J, Obara T, . Surveys of postpartum depression in Miyagi, Japan, after the Great East Japan Earthquake. Arch Women Ment Health. 2014;17:579–581. 10.1007/s00737-014-0459-y 25204487 PMC4237927

[27] Sato K, Oikawa M, Hiwatashi M, Sato M, Oyamada N. Factors relating to the mental health of women who were pregnant at the time of the Great East Japan earthquake: analysis from month 10 to month 48 after the earthquake. Biopsychosoc Med. 2016;10:22. 10.1186/s13030-016-0072-6 27354853 PMC4924283

[28] Yoshida H, Hayashi K, Ohta H, . Perinatal outcome and mother and child support project in the acute phase of the Great East Japan Earthquake. J Jpn Prim Care Assoc. 2015;38:136–141 [in Japanese].

[29] Nishigori H, Sasaki M, Obara T, . Correlation between the Great East Japan Earthquake and postpartum depression: a study in Miyako, Iwate, Japan. Disaster Med Public Health Prep. 2015;9:307–312. 10.1017/dmp.2015.51 25896395

[30] Harville EW, Xiong X, Smith BW, . Combined effects of Hurricane Katrina and Hurricane Gustav on the mental health of mothers of small children. J Psychiatr Ment Health Nurs. 2011;18:288–296. 10.1111/j.1365-2850.2010.01658.x 21418428 PMC3472438

[31] Tees MT, Harville EW, Xiong X, . Hurricane Katrina-related maternal stress, maternal mental health, and early infant temperament. Matern Child Health J. 2010;14:511–518. 10.1007/s10995-009-0486-x 19554438 PMC3472436

[32] Ehrlich M, Harville E, Xiong X, . Loss of resources and hurricane experience as predictors of postpartum depression among women in southern Louisiana. J Womens Health (Larchmt). 2010;19:877–884. 10.1089/jwh.2009.1693 20438305 PMC2875990

[33] Harville EW, Xiong X, Buekens P, Pridjian G, Elkind-Hirsch K. Resilience after hurricane Katrina among pregnant and postpartum women. Womens Health Issues. 2010;20:20–27. 10.1016/j.whi.2009.10.002 20123173 PMC2822707

[34] Demirchyan A, Petrosyan D, Armenian HK. Rate and predictors of postpartum depression in a 22-year follow-up of a cohort of earthquake survivors in Armenia. Arch Women Ment Health. 2014;17:229–237. 10.1007/s00737-013-0404-5 24435250

[35] Goto A, Rudd RE, Bromet E, . Maternal confidence of Fukushima mothers before and after the nuclear power plant disaster in Northeast Japan: analyses of municipal health records. J Commun Healthc. 2014;7:106–116. 10.1179/1753807614Y.0000000051

[36] Radiation Medical Science Center for the Fukushima Health Management Survey. Materials and minutes of prefectural oversight committee meetings. 35th report (July 8, 2019). http://kenko-kanri.jp/en/health-survey/document/pdf/35_8July2019.pdf; 2019 Accessed 07.17.2021.

[37] Sato K. The impact of the Great East Japan Earthquake on the mental health of mothers. Jpn J Midwives. 2012;66:858–863 [in Japanese].

[38] Ren JH, Chiang CLV, Jiang XL, . Mental disorders of pregnant and postpartum women after earthquakes: a systematic review. Disaster Med Public Health Prep. 2014;8:315–325. 10.1017/dmp.2014.62 25098648

[39] Qu Z, Wang X, Tian D, . Posttraumatic stress disorder and depression among new mothers at 8 months later of the 2008 Sichuan earthquake in China. Arch Womens Ment Health. 2012;15:49–55. 10.1007/s00737-011-0255-x 22249399

[40] Brock RL, O’Hara MW, Hart KJ, . Partner support and maternal depression in the context of the Iowa floods. J Fam Psychol. 2014;28:832–843. 10.1037/fam0000027 25243576 PMC4277699

[41] Levi R, Lundberg U, Hanson U, Frankenhacuser M. Anxiety during pregnancy after the Chernobyl accident as related to obstetric outcome. J Psychosom Obstet Gynecol. 1989;10:221–230. 10.3109/01674828909016696

[42] Kolominsky Y, Igumnov S, Drozdovitch V. The psychological development of children from Belarus exposed in the prenatal period to radiation from the Chernobyl atomic power plant. J Child Psychol Psychiatry. 1999;40:299–305. 10.1111/1469-7610.00444 10188713

[43] Remennick LI. Immigrants from Chernobyl-affected areas in Israel: the link between health and social adjustment. Soc Sci Med. 2002 Jan;54(2):309–317. 10.1016/S0277-9536(01)00030-2 11824934

[44] Arosemena FA, Fox L, Lichtveld MY. Reproductive Health Assessment After Disasters: embedding a toolkit within the disaster management workforce to address health inequalities among Gulf-Coast women. J Health Care Poor Underserved. 2013;24(4 Suppl):17–28. 10.1353/hpu.2014.0013 24241257

[45] Savage J, Giarratano G, Bustamante-Forest R, . Post-Katrina perinatal mood and the use of alternative therapies. J Holist Nurs. 2010;28:123–132. 10.1177/0898010109348388 20522707

[46] Kildea S, Simcock G, Liu A, . Continuity of midwifery carer moderates the effects of prenatal maternal stress on postnatal maternal wellbeing: the Queensland flood study. Arch Women Ment Health. 2018;21:203–214. 10.1007/s00737-017-0781-2 28956168

[47] Giarratano G, Harville EW, Barcelona de Mendoza V, . Healthy start: description of a safety net for perinatal support during disaster recovery. Matern Child Health J. 2015;19:819–827. 10.1007/s10995-014-1579-8 25047787 PMC4303559

